# Pan-cancer analysis of intratumor heterogeneity as a prognostic determinant of survival

**DOI:** 10.18632/oncotarget.7067

**Published:** 2016-01-28

**Authors:** Luc G.T. Morris, Nadeem Riaz, Alexis Desrichard, Yasin Şenbabaoğlu, A. Ari Hakimi, Vladimir Makarov, Jorge S. Reis-Filho, Timothy A. Chan

**Affiliations:** ^1^ Human Oncology and Pathogenesis Program, Memorial Sloan Kettering Cancer Center, New York, NY, USA; ^2^ Department of Surgery, Memorial Sloan Kettering Cancer Center, New York, NY, USA; ^3^ Department of Radiation Oncology, Memorial Sloan Kettering Cancer Center, New York, NY, USA; ^4^ Computational Biology Center, Memorial Sloan Kettering Cancer Center, New York, NY, USA; ^5^ Department of Pathology, Memorial Sloan Kettering Cancer Center, New York, NY, USA

**Keywords:** heterogeneity, evolution, survival, cancer, immune surveillance

## Abstract

As tumors accumulate genetic alterations, an evolutionary process occurs in which genetically distinct subclonal populations of cells co-exist, resulting in intratumor genetic heterogeneity (ITH). The clinical implications of ITH remain poorly defined. Data are limited with respect to whether ITH is an independent determinant of patient survival outcomes, across different cancer types. Here, we report the results of a pan-cancer analysis of over 3300 tumors, showing a varied landscape of ITH across 9 cancer types. While some gene mutations are subclonal, the majority of driver gene mutations are clonal events, present in nearly all cancer cells. Strikingly, high levels of ITH are associated with poorer survival across diverse types of cancer. The adverse impact of high ITH is independent of other clinical, pathologic and molecular factors. High ITH tends to be associated with lower levels of tumor-infiltrating immune cells, but this association is not able to explain the observed survival differences. Together, these data show that ITH is a prognostic marker in multiple cancers. These results illuminate the natural history of cancer evolution, indicating that tumor heterogeneity represents a significant obstacle to cancer control.

## INTRODUCTION

Many of the somatic mutations found in cancer are clonal events, which occur in the founding cell at the time of tumor initiation, and are then propagated during clonal expansion. The model of branching evolution in cancer proposes that these “trunk mutations” are found in every cancer cell comprising a solid tumor. Subsequent “branch mutations” are subclonal events that only exist in a subpopulation of tumor cells. These subclonal populations of cells will expand or regress in response to the selective pressures exerted by the tumor microenvironment and cancer-directed treatment [1, 2]. Altogether, the repertoire of admixed clonal and subclonal populations is referred to as intratumor genetic heterogeneity (ITH).

Multifaceted studies of cancer genomes now allow the identification of clonal and subclonal alterations within tumors. Initial studies employed multi-sample sequencing to characterize genetic heterogeneity across different regions of a tumor [3-6], or single-cell sequencing [7-9], to define ITH within an individual cancer case. However, next-generation sequencing data derived from even single regions, or “bulk” tumor material can be analyzed to uncover subclonal populations. This approach has been shown to provide sufficient resolution for a window into a tumor's overall level of heterogeneity, and could allow for the study of larger numbers of tumors [10-16].

It has been hypothesized that ITH would be associated with poorer clinical outcomes in cancer patients, but supportive evidence to date has been limited to small series in individual cancer types. This has mainly been studied in specific contexts, such as resistance to therapy. For example, as treatment with chemotherapy, radiotherapy, or targeted agents applies selective pressure on a population of subclones, certain subpopulation(s) with resistance mutations can be observed to expand and populate recurrent tumors [17-21]. A key question is whether ITH has independent impact on a tumor's natural history or a patient's outcome, beyond known clinical, pathologic and molecular factors.

More generally, we hypothesize that the degree of ITH in a tumor can be considered an indication of a tumor's potential for evolutionary adaptation. If this is true, we reason that ITH would be associated with more aggressive tumor behavior and poorer patient survival. The strongest evidence for this comes from a study of head and neck cancers, in which the degree of variation in mutational allelic frequencies was associated with poorer outcome [22].

To date, there have not been any large-scale studies investigating whether ITH is prognostic for patients with different types of cancer, providing information beyond standard clinicopathologic prognostic factors. If prognostic, ITH would have value akin to clinical staging, which can predict a patient's likely clinical outcome at the time of initial diagnosis. Here, our objective was to determine whether ITH, analyzed in tumors at the time of treatment, impacts clinical outcome in patients. To this end, we performed a pan-cancer analysis of 9 cancer types (in total, 3383 tumors), integrating measures of tumor genetic heterogeneity with multifaceted clinical, pathologic and molecular data, to define the impact of ITH on patient survival.

## RESULTS

### Patient and tumor characteristics

**Figure 1:**
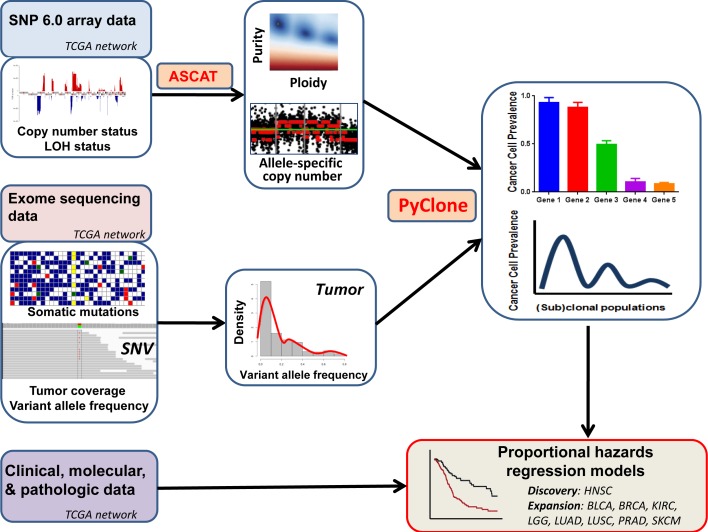
Analysis of intratumor heterogeneity, and its prognostic significance, using multifaceted genomic data SNP6 array data are used to determine allele-specific copy number and tumor purity. These data are integrated with mutation variant allele frequencies in exome sequencing data to infer the cellular prevalence of each mutation. (Sub)clonal populations are identified by clustering mutations by their cellular prevalence. Multivariable models of survival can then be generated, incorporating intratumor heterogeneity and other relevant clinical factors.

To measure intratumor genetic heterogeneity (ITH) in cancers, we analyzed data from studies performed by The Cancer Genome Atlas Network (TCGA), for which 3 types of complete data were available for >100 tumors: 1) SNP 6.0 array copy number, 2) single nucleotide variant read counts, and 3) clinical patient and tumor data (Figure 1). TCGA tumors are high quality, frozen samples that are generally obtained from surgical resections. Clinical data are updated at regular intervals by the TCGA in concert with treating clinicians. We did not include cancer types if cohort sample sizes were <100, if these 3 data types were not publicly available in full, or if the cancer type exhibited a narrow range in survival times (e.g., glioblastoma or papillary thyroid carcinoma). We analyzed data for nine cancer sites: bladder urothelial carcinoma (BLCA, *n* = 359), breast invasive carcinoma (BRCA, *n* = 878), head and neck squamous cell carcinoma (HNSC, *n* = 280), clear cell carcinoma of the kidney (KIRC, *n* = 189), lower grade glioma (LGG, *n* = 484), lung adenocarcinoma (LUAD, *n* = 425), lung squamous cell carcinoma (LUSC, *n* = 178), prostate adenocarcinoma (PRAD, *n* = 389), and melanoma (SKCM, *n* = 201). Altogether these data comprised 3383 tumors with genetic and clinical data. We report the results of all analyzed datasets, and this study adheres to the REMARK (Reporting Recommendations for Tumor Marker Prognostic Studies) reporting guidelines [23] (*see Methods*).

### Landscape of subclonal populations

The analysis workflow is depicted in Figure 1. For each primary tumor, we used SNP 6.0 array data to determine allele-specific copy number with ASCAT (allele-specific copy number of tumors), which estimates and adjusts for ploidy and non-variant cell admixture [24]. We validated the copy number-based purity estimates with an orthogonal expression-based technique, which produced highly concordant results (*p* = 1×10^−7^; see *Methods*) [25]. Using the exome data and TCGA somatic mutation calls, we obtained measures of tumor coverage and variant allele frequency for each single nucleotide variant. For each tumor, we input aberrant cell fraction and major and minor allele copy number from ASCAT, together with variant allele frequencies for somatic mutations, into PyClone [14]. PyClone estimates the cancer cell prevalence for each somatic mutation in a sequenced tumor sample, and subsequently uses Bayesian methodology to cluster cancer cell prevalence estimates into clonal populations. Intratumor heterogeneity can be expressed as the number of genetically distinct (sub)clonal populations (SCPs) in a tumor, where each SCP represents the outcome of a clonal expansion. We included SCPs containing ≥2 mutations, because SCPs defined by only 1 mutation are of unknown biologic significance. Because these tumors are only sampled in a single region, these measurements of SCPs may underestimate geographic ITH across regions of a tumor. However, comparable methodologies integrating purity, ploidy and mutational read counts to identify SCPs have been used in prior studies [10-12, 15, 16, 26, 27] and have been shown to have high concordance with single-cell sequencing data [9] and histologic measures of ITH [15]. This study therefore evaluates the prognostic importance of ITH observed in single-region tumor sequencing. As an orthogonal confirmation of our measurements of ITH, we separately inferred SCPs with an independent tool (EXPANDS), which also determines subclonal architecture by integrating somatic mutation allele frequencies with local ploidy, albeit with a different mathematical model [16]. EXPANDS and PyClone-based analyses have been shown to be highly concordant[15], and were strongly correlated in our primary analysis (*p* = 3.01 x10^−4^; see *Methods*).

**Figure 2:**
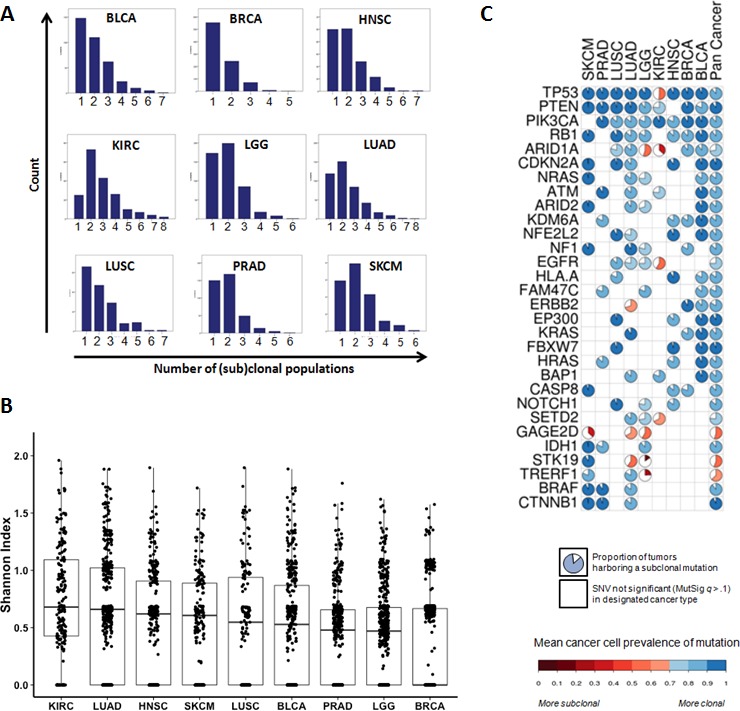
The landscape of intratumor heterogeneity across different types of cancer **A.** Histograms of the distribution of (sub)clonal populations in 9 cancer types. **B.** Boxplots of intratumor heterogeneity, expressed as the Shannon Index of diversity, by cancer type. The Shannon Index approaches zero as the number of (sub)clonal populations decreases, or become more equal in size. **C.** Spectrum of clonality among recurrently mutated (MutSig q<0.10, present in ≥3 cancer types) genes. Each pie is colored by the mean cancer cell prevalence of the gene mutation in that cancer type. The size of the white pie slice indicates the proportion of mutations categorized as subclonal (<70% cellular prevalence). For a more comprehensive survey of genes, see Supplementary Figure 1.

The landscape of SCPs across 9 cancer types is depicted in Figure 2A. SCPs were identified in 3364 (99.4%) tumors. These data were derived from tumors processed by the Biospecimen Core Resource of the TCGA, which extracts significant amounts of resected tissue from each tumor. Although the goal of such processing is not to separate distinct parts of the tumor, the processing is likely to provide a broad representation of tumor DNA. Based on these datasets, the mean number of SCPs was 1.96. The majority of bladder, breast, and lung squamous cancers were dominated by a single clonal expansion, while most head and neck, kidney, lower grade glioma, lung adenocarcinoma, prostate and melanoma tumors were clearly polyclonal. Overall, 59% of tumors were polyclonal, and 24% harbored more than 2 clonal populations (Supplementary Table 1). ITH was lowest in breast cancers, where the vast majority of tumors (63.1%) had one dominant clonal population, with mean SCP number 1.47. This distribution is similar to findings reported in an independent breast cancer study [12], and in a study of several other cancer types [15]. Heterogeneity was highest in clear cell renal carcinoma, where 13.2% of tumors were defined by only one clonal expansion, and mean SCP number was 2.84. ITH can also be compared in a more granular fashion, across tumors and cancer types, using the Shannon index, which is a measure of information uncertainty that is commonly used to quantify subpopulation diversity (Figure 2B, Supplementary Table 2).

For each cancer type, the majority of MutSig significantly (*q*<.10) mutated genes had SNVs with mean cancer cell prevalence (CCP) >90%. This indicates that most recurrent cancer gene mutations are present in every cancer cell. This is consistent with a scenario where these clonal mutations result from early events in tumorigenesis. For example, frequent mutations in driver genes such as *TP53, RB1*, *CTNNB1, KEAP1, MLL2, PTEN* and *CDKN2A* were almost universally clonal events (>90% mean CCP). However, there were some notable exceptions to this pattern of clonal mutations in driver genes, such as *IDH1* in LGG (88% mean CCP), *EGFR* in LUAD (78%), and *PIK3CA* in HNSC (83%), LUAD (86%) and LUSC (87%). These results are largely concordant with findings reported in a prior study [11]. Interestingly, we observed that many lower-prevalence mutations in driver genes were often subclonal. These included *ERBB2* in LUAD (70% mean CCP), *NFE2L2* in LUAD (78%, compared to 92% in LUSC), *ARID1A* in lower grade glioma (55%), and multiple genes in KIRC (*PTEN*, 77%; SETD2, 68%; *TP53*, 51%; *ARID1A*, 37%). In fact, nearly all mutated genes in clear cell renal carcinoma, which had the highest overall degree of ITH, were commonly subclonal (Figure 2C and Supplementary Figure 1).

### Intratumor heterogeneity and clinical outcome

To dissect the relationship between ITH and clinical outcome, we first examined head and neck squamous cell carcinoma (HNSC). This tumor type was chosen due to published data suggesting an association of ITH with survival. This prior analysis [22, 28] used a measure of dispersion in variant allelic frequencies called MATH. Analyzing clinical, pathologic and genetic data from 280 tumors in the TCGA HNSC dataset, we confirmed that a number of known prognostic factors were associated with overall survival (OS), including clinical stage (tumor/node/metastasis), human papillomavirus (HPV) status, *TP53* mutation, degree of copy number alteration and mutational load, and predominance of copy number alteration (C class) or mutations (M class) [29] (Supplementary Figure 2). We also confirmed that higher MATH scores were associated with poorer OS in HNSC (Supplementary Figure 3). Perhaps because MATH is based on variability in mutation allelic frequencies, MATH scores were most strongly associated with each tumor's degree of copy number alteration (*p* < 0.001), moreso than with measures of (sub)clonal populations (*p* = 0.35) (Supplementary Figure 4, Supplementary Table 3).

**Figure 3:**
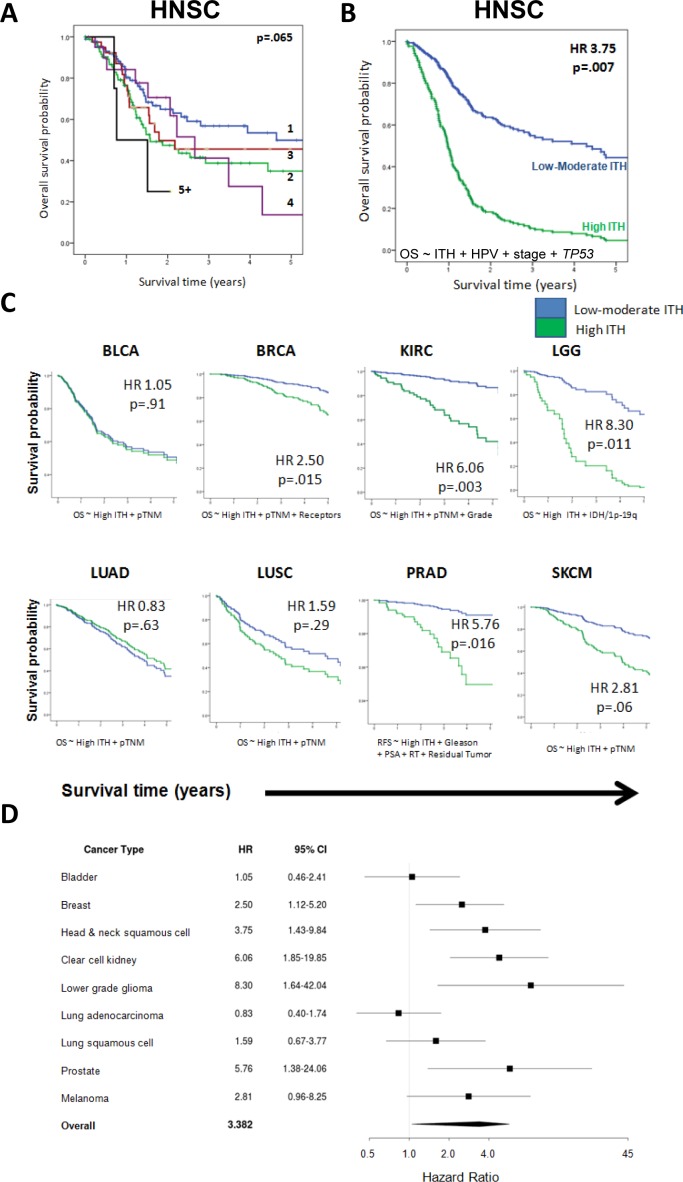
Intratumor heterogeneity is associated with poorer patient survival **A.** Kaplan-Meier curve of overall survival by degree of intratumor heterogeneity in the discovery dataset (HNSC). A trend toward poorer overall survival is evident as the number of subclonal populations increases. The log-rank test was used for comparisons. **B.** Overall survival for low and high ITH HNSC tumors, adjusting for HPV status, stage, and TP53 mutation status. The survival curve is plotted at the mean of other covariates in the multivariable model. **C.** Survival curves for low and high ITH tumors in 8 additional cancer types. In each case, curves are plotted at the mean of other covariates adjusted for in the regression models. The x-axis ranges from 0-5 years. The y-axis depicts the probability of overall survival, except for PRAD, where relapse-free survival is used. **D.** Hazard ratios for death (relapse in PRAD), with 95% confidence intervals, for each cancer type, where ITH is adjusted for other covariates as shown in **C.**

In HNSC, we observed a consistent trend of decreasing OS with increasing heterogeneity. (HR for polyclonal tumors=1.23, *p* = .033; Figure 3A). The association was strongest for high levels of ITH (“High ITH,” SCP>4 or z-score of log-transformed SCP +1.75; HR = 2.91, *p* = .022, Supplementary Table 4). Starting with all relevant covariates (to a limit of one covariate per 15 events), we used stepwise Cox multivariable regression to build a parsimonious model for overall survival, incorporating ITH and all of the above clinical, pathologic and molecular prognostic factors. This demonstrated that high ITH was independently associated with OS (HR = 2.51; *p* = .007), when adjusting for HPV status, *TP53* mutation, and stage (Figure 3B, Supplementary Table 5A). In contrast to ITH, other genomic metrics (such as mutational load and MATH) did not have independent prognostic value when controlling for these same covariates (Supplementary Table 5B-C). The association between high ITH and OS was independent of adjuvant radiotherapy administration (Supplementary Table 5D, Supplementary Figure 5), indicating that ITH reflects prognostic aspects of tumor biology, rather than simply response to therapy.

Having established that high ITH had prognostic value in HNSC, we then asked if this factor was informative in the 8 other cancer types. We decided *a priori* to apply the same SCP thresholds for high ITH as in HNSC (SCP > 4 and z-score +1.75; see *Methods*). For each cancer type, we examined the association between ITH and OS in multivariable analyses, controlling for any clinical, pathologic and molecular factors that were significant on univariate analysis or clinically relevant. For prostate cancer (PRAD), we modeled relapse-free survival (RFS), because deaths were rare during the available follow-up period. We found that high ITH was associated with significantly poorer survival in LGG (HR = 8.30, *p* = .011), PRAD (HR = 5.76, *p* = .016), KIRC (HR = 6.06, *p* = .003), HNSC (HR = 3.75, *p* = .007), and BRCA (HR = 2.50, *p* = .015). These 5 associations remained significant controlling false discovery rate (Benjamini-Hochberg) at *q* < .10. There was a borderline significant trend toward poorer OS in SKCM (HR = 2.81, *p* = .06). No association was observed in LUSC (HR = 1.59, *p* = .29), BLCA (HR = 1.05, *p* = .91) and LUAD (HR = 0.83, *p* = .63). The results of these analyses are summarized in Figure 3C-3D. Complete parameters of all multivariable models are detailed in the Table 1.

**Table 1:** Multivariable Cox regression models for overall survival in all cancer types, incorporating ITH and other covariates

Cancer Type	Survival outcome	Covariates	HR	95% CI	p
**Bladder urothelial**	Overall survival	High ITH	1.05	0.46-2.41	0.91
		Stage	-	-	0.001
**Breast**	Overall survival	High ITH	2.50	1.12-5.20	0.015
		Stage	-	-	<.001
		Receptor status*	-	-	0.002
		*ER+/Her2-, ER+/Her2+, ER-/Her2+, Triple Negative			
	Overall survival	High ITH	2.44	1.05-5.69	0.039
		Stage	-	-	0.013
		PAM50 subtype*	-	-	0.11
		* Basal, Luminal A, Luminal B, Her2-enriched, Normal-like			
**Head & neck squamous cell**	Overall survival	High ITH	3.75	1.43-9.84	0.007
		HPV-negative	2.51	1.13-5.59	0.024
		TP53 mutated	1.55	1.03-2.33	0.038
		Stage	-	-	0.304
**Clear cell renal**	Overall survival	High ITH	6.06	1.85-19.85	.003
		Stage	-	-	0.001
		Grade	-	-	0.145
**Lower grade glioma**	Overall survival	High ITH	8.30	1.64-42.04	0.011
		IDH, 1p-19q status*	-	-	<.001
		*IDH mutant vs WT; 1p-19q co-deleted vs normal			
**Lung adenocarcinoma**	Overall survival	High ITH	0.83	0.40-1.74	0.63
		Stage	-	-	<.001
**Lung squamous cell**	Overall survival	High ITH	1.59	0.67-3.77	0.29
		Stage	-	-	0.87
**Prostate**		High ITH	5.76	1.38-24.06	0.016
		Gleason score	1.35	0.82-2.24	0.25
	Relapse-free survival	PSA level	1.06	1.02-1.11	0.005
		Stage	-	-	0.97
		Adjuvant RT	0.94	0.28-3.11	0.9
		Resection margin	-	-	0.38
**Melanoma**	Overall survival	High ITH	2.81	0.96-8.25	0.06
		Stage	-	-	0.015

### Intratumor heterogeneity and molecular subtypes

High levels of ITH may be associated with poorer outcome as the likelihood of treatment-resistant subclonal populations increases. ITH may also be considered an indicator of a tumor's potential to adapt by undergoing evolution under selective pressure. We performed additional analyses to rule out the possibility that high ITH was merely a surrogate marker for other clinical or molecular factors driving poorer prognosis.

**Figure 4:**
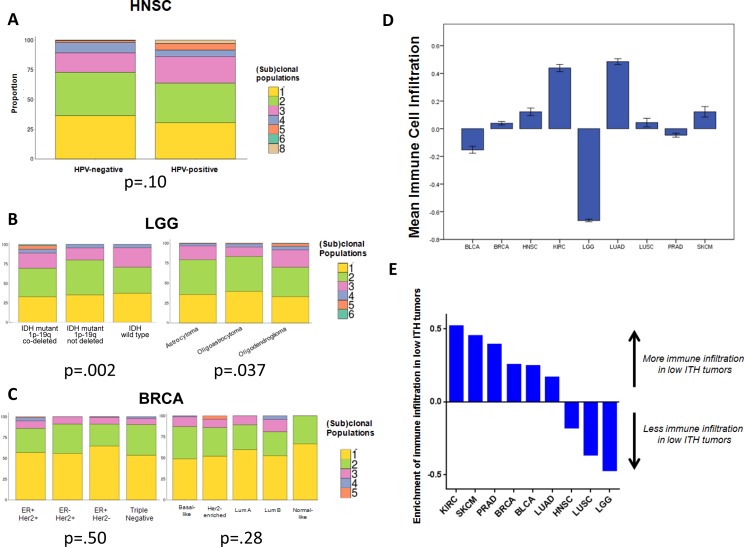
Associations of intratumor heterogeneity with other clinical, molecular, and immune factors **A.** Distribution of (sub)clonal populations by HPV status in HNSC, showing a non-significant trend toward higher ITH in HPV+ tumors. **B.** Distribution of (sub)clonal populations by molecular and histologic subtype in LGG. High ITH was more common in *IDH* mutant, 1p-19q co-deleted tumors, and in oligodendrogliomas. **C.** Distribution of (sub)clonal populations by molecular and receptor subtype in BRCA, showing no differences between subgroups. Categorical comparisons were made with the Fisher exact or χ^2^ test. **D.** Mean levels of RNAseq-derived immune cell infiltration, by cancer type. Error bars represent ± 1 SEM. **E.** Column graph showing enrichment for immune cell infiltration in tumors with low ITH, by cancer type. The y-axis represents the z-score of absolute increase, or decrease, in immune infiltration in low ITH tumors.

The Cox regression models for survival included adjustment for multiple factors, including prognostically-relevant molecular subtypes, which have relevance in tumor types such as head and neck cancer, breast cancer, and lower grade glioma. We performed additional analyses to determine if tumors with high ITH had poorer prognosis due to an association with more aggressive molecular subtypes, and found that this was not the case. For HNSC, the degree of heterogeneity was slightly higher among HPV+ tumors (independent sample t-test *p* = .10; Figure 4A), a subtype with superior survival (Supplementary Figure 2A). Among LGGs, ITH was strongly prognostic, even when controlling for IDH mutation and 1p-19q co-deletion status. The majority of high ITH tumors were IDH-mutant/1p-19q co-deleted (chi-squared *p* = .002) or had oligodendroglioma histology (chi-squared *p* = .037) (Figure 4B). This subtype has the best prognosis among LGGs (Supplementary Figure 6A). Within this category of tumors, high ITH remained associated with poorer survival (*p* = .01) (Supplementary Figure 6B). In the BRCA cohort, we found that high ITH was associated with poorer survival, independent of clinical stage and receptor status. PAM50 expression-derived molecular subtype data were available for 522 of 878 cases; when substituting PAM50 subtype for receptor status, high ITH remained a significant prognostic factor (HR = 2.44, *p* = .03) (Supplementary Figure 7A). High ITH was not more common in poorer-prognosis tumors such as triple negative receptor status or basal-like molecular subtype. In fact, high ITH status was evenly distributed across receptor (*p* = .50) and PAM50 (*p* = .28) subtypes (Figure 4C). The only enrichment was seen in the very highest ITH tumors (top 1%), which were concentrated in the ER+/Her2+ (*p* = .012) receptor subtype and the Luminal B (*p* = .043) molecular subtype. The prognostic impact of high ITH appeared to affect all molecular subtypes but was most significant in the Her2-enriched tumors (*p* = .04) (Supplementary Figure 7B).

### Intratumor genetic heterogeneity and other factors

We then examined other clinical or genetic factors for correlation with ITH. As would be expected, the degree of ITH was strongly correlated with mutation number in most cancer types - all except LUAD and LUSC (Supplementary Table 6A). Mutational load was added as a covariate in all Cox regression models, to examine whether this factor explained the prognostic value of high ITH. In all cancer types, ITH, but not mutational load, was prognostically significant (Supplementary Table 6B). This indicates that the prognostic value of ITH is not mediated by any prognostic impact of mutational load. In fact, any prognostic aspect of mutational load in tumors could be mediated by ITH.

High ITH was associated with older age in 1 of 9 cancer types - LGG (*p* = .001) (Supplementary Table 7A). We performed additional Cox multivariable modeling to adjust for age, and found that ITH still retained independent prognostic value in LGG (HR for high ITH = 10.19, *p* = .005) (Supplementary Table 7B). We did not identify any other significant associations between ITH and additional patient or tumor characteristics, including tumor size, disease stage, smoking history, prior treatment, subsequent treatment, or genetic alterations.

### Immune infiltration and tumor heterogeneity

We also considered that ITH might be associated with altered levels of tumor-infiltrating immune cells, which could be driving the observed survival differences. Recent data in melanoma and non-small cell lung cancer have shown that tumors with a higher mutational burden have an increased number of predicted neoantigens, and are more likely to respond to immunotherapy [30, 31]. It is believed that the neoantigens resulting from tumor mutations promote anti-tumor immunity and thereby facilitate therapeutic responses. We therefore asked if more genetically heterogeneous tumors had higher levels of immune cell infiltration. To this end, we analyzed RNAseq data to measure immune populations in the microenvironment of sequenced tumors [32] (see *Methods*). This approach infers the relative infiltration levels of most immune cells and overexpression of genes related to IFNγ signaling (Figure 4D, Supplementary Figure 8A-8B).

Across all cancers, we found an inverse association between tumor heterogeneity and immune cell infiltration. Tumors with high ITH tended to have lower levels of immune cell infiltration, or T cell infiltration, controlling for cancer type with logistic regression (immune infiltration *p* = .020, T cell infiltration *p* = .055) (Supplementary Table 8; Supplementary Figure 8C-E). When stratified by cancer type, there was insufficient statistical power to discern differences in all subsets, but a similar numerical trend was recapitulated in the majority of cancer types (Figure 4E, Supplementary Figure 8C-D). The association was most statistically significant for clear cell renal carcinoma (KIRC), which showed markedly elevated immune infiltrates in low ITH tumors (OR = 0.23, p=.009). We therefore performed additional multivariable survival modeling in KIRC, including immune infiltrate data as a covariate, and found that this did not attenuate the prognostic strength of ITH (Supplementary Table 9A). Likewise, for HNSC, where the reverse trend was seen - immune and T cell infiltrates were elevated in high ITH tumors - we found that immune infiltrate data did not attenuate the prognostic strength of ITH (Supplementary Table 9B). Taken together, these data indicate that, while ITH is associated with the level of immune cell infiltrates, immune infiltration does not mediate the poorer survival associated with high levels of ITH.

## DISCUSSION

Intratumor genetic heterogeneity (ITH) is a feature of tumors that refers to the repertoire of co-existing genetically distinct subclonal populations. ITH is believed to result from a process of branching evolution. Mathematical models reveal that some degree of ITH is probably present in all large solid tumors [33]. The expansion of subclonal populations under selective pressure is believed to explain the phenomenon of resistance to targeted cancer therapy [17, 19-21, 34]. However, beyond the context of predicting tumor responses to specific therapies, data are limited with respect to whether intratumor heterogeneity has prognostic value for patient survival, independent of existing clinical and pathologic factors.

Here, in a pan-cancer analysis of over 3300 tumors, we demonstrate that genetically heterogeneous tumors, comprised of multiple subclonal populations, tend to be associated with poorer patient survival than tumors harboring low/moderate levels of intratumor heterogeneity. We found that the prognostic value of ITH was significant in many cancer types, and in multivariable analyses, remained significant when controlling for other relevant clinical, pathologic and molecular features.

Our analyses integrated SNP array copy number and whole exome sequencing mutational data from TCGA datasets, to infer the subclonal architecture of tumors, and to classify them as having low or high degrees of intratumor heterogeneity. These genomic data were obtained from sequencing of single tumor regions. Therefore, these analyses may potentially understate the wider degree of heterogeneity across different spatial regions in a tumor. Nevertheless, we found that this measure of ITH was sufficiently sensitive to be strongly associated with survival outcome in multiple cancer types. The association between high ITH and poorer survival was most evident in lower grade glioma, prostate, clear cell kidney, head and neck, and breast cancers, and had borderline significance in melanoma. We applied consistent definitions of high ITH to each of these different cancers, and observed similar effects on survival outcomes.

It is possible that a more sensitive measure of ITH, such as multi-region or single cell sequencing, would have stronger prognostic value in additional cancer types. Nevertheless, it is particularly compelling for a molecular or genetic biomarker to have significant prognostic value across diverse types of cancer, suggesting that an important aspect of tumor biology can be captured in the measurement of ITH within single samples.

These results, showing the independent prognostic value of ITH, are consistent with hypotheses and data that have been generated by studies within individual cancer types. For example, in a study of patients with relapsed chronic lymphocytic leukemia, subclonal mutations were associated with more aggressive disease [27]. Similar findings were observed in 11 patients with lung adenocarcinoma: recurrent tumors had more subclonal mutations [35]. In a series of head and neck cancers, the degree of dispersion in mutation allelic frequencies was linked with survival outcome, showing the prognostic value of this metric in HNSC, and suggesting that tumors with more subclonal populations may have poorer outcome [22]. Limitations of these prior studies include differing sets of methods used to define ITH, and limited patient numbers. Our findings expand on this prior work, by applying a unified methodology to measure ITH, across a large set of tumors representing a diverse set of cancers.

In a pan-cancer study examining the landscape of ITH, Andor *et al* did not observe a clear association between ITH and survival within individual cancers, but did observe that prognosis appeared poorest among tumors with intermediate levels of copy number variation. Across all tumor samples, survival appeared to be better among tumors with lower levels of ITH, but this association was confounded by comparing low ITH cancer types (thyroid and prostate - cancers with low mortality) to high ITH tumor types (melanoma and lung - cancers with higher mortality)[15]. Altogether, these preliminary data have generated a strong rationale to systematically explore the prognostic significance of ITH. The question of whether ITH provides truly prognostic information in cancer, above and beyond clinical, pathologic and molecular factors, had heretofore remained unanswered.

In this study, we first examined subclonal populations in HNSC, a cancer type in which prior data suggested an association between ITH and clinical outcome. We identified a prognostic cutpoint for ITH, and applied this same threshold to 8 other cancer types, using multivariable regression to adjust for other relevant clinicopathologic factors. To rule out the possibility that ITH was not *per se* prognostic, and might merely be acting as a surrogate marker of another prognostic variable, we examined the association between ITH and multiple other factors, such as age, extent of disease, tumor size, molecular subtype, genetic features, smoking exposure, and types of prior or subsequent treatments. ITH and mutational burden were strongly correlated, but multivariable models demonstrated that ITH, and not mutation number, was the prognostic factor.

We also noted a tendency for high ITH tumors to have lower levels of immune infiltration. This relationship was strongest in clear cell renal carcinoma and melanoma, cancer types in which immune surveillance is known to be functional. Additional multivariable models showed that the prognostic impact of ITH was not mediated by these differences in immune infiltration.

These associations are potentially consistent with the model of cancer immunoediting, a dynamic process proposed by Schreiber, in which the immune system sculpts the formation of cancers by eliminating (or “editing”) immunogenic components of a tumor. Immunoediting has been shown to be largely mediated by T-cell recognition of tumor antigens, leading to the selective outgrowth of tumor subclones that lack rejection antigens and have lower levels of immunogenicity [36]. This mechanism has been recently observed in the control of tumor ploidy. An intact immune system, via T lymphocytes and interferons, is able to selectively eliminate hyperploid cancer cells, in contrast to their diploid counterparts [37]. This raises the possibility that the immune system might also constrain subclonal evolution within tumors. Indeed, our findings suggest that low ITH tumors may, in part, reflect the results of immunoediting; specifically, the elimination of certain immunogenic subclonal populations. Because the ITH analysis adjusts for tumor purity, this association does not reflect the effects of immune infiltration on mutational frequencies. However it is important to note that these data are only hypothesis-generating. A genomic analysis cannot conclusively demonstrate this process, since the key subclonal populations in question are absent. We cannot conclude whether they were eliminated by the immune system, or were never there to begin with. However, if immunoediting can in fact constrain ITH, one could speculate that the cancers most likely to benefit from immunotherapy would be those with high mutational loads (and thus, many neoantigens), but low ITH (thereby, having undergone more immunoediting by a functional immune surveillance system). Further work will be necessary to explore the relationship between immune surveillance, immunoediting, and subclonal populations.

The vast majority of highly prevalent driver mutations tended to be clonal events, present in every cancer cell, suggestive that these are generally early or “trunk” mutational events. In contrast, some lower prevalence mutations in driver genes (such as *TP53* in KIRC, or *NFE2L2* in LUAD) tended to be mostly subclonal. These findings provide empirical evidence for recent theoretical models of tumor evolution, showing that driver mutations with even a small fitness advantage are able to expand and become dominant within a short period of time, whereas passenger mutations without a major fitness advantage are more likely to remain subclonal [38]. However, there are some important exceptions to this pattern. We found that clear cell kidney cancers tend to have high degrees of ITH, and therefore, most driver genes in this cancer type were subclonal. Furthermore, in other cancer types, some prevalent driver gene mutations appeared to be frequently subclonal. This phenomenon has also been observed in a prior study, which reported that 15-20% of mutations in driver genes such as *IDH1*, *PIK3CA* and *EGFR* were subclonal, and linked many of these to a mutational signature of APOBEC-mediated mutagenesis [11].

There are several caveats to our analysis. First, ITH was delineated in single tumor samples, and limited to mutations (not including subclonal structural variants or copy number alterations). Statistical power was also limited by sequencing depth, reducing sensitivity for low frequency subclonal events (such as those occurring below 5-10% variant allele frequency) [39]. These limitations would tend to attenuate the strength of associations in this study: the measures of ITH in this study are likely to be conservative, potentially underestimating the true extent of heterogeneity in tumors, as evidenced by studies that have sequenced multiple regions of tumors [3-5, 40, 41]. We do note that other prior studies have used similar methodology to our study [10-12, 15, 16], and that single-sample analyses of ITH using PyClone or EXPANDS have been shown to correlate strongly with histologic and cellular measures of ITH [15]. Therefore, these analytic techniques have been shown to be sufficiently sensitive to generate informative ITH data. To generate a truly exhaustive picture of ITH may require sequencing at the single cell level [7, 42]. Given these limitations, our study is likely to underestimate the prognostic value of ITH which may in fact be greater, and more generalizable to other cancer types, than what we were able to discern.

On the other hand, an advantage to the exploration of ITH in single samples is scale, an important requirement when performing multivariable analyses of clinical biomarkers. A statistical strength of this study was the inclusion of over 3000 tumors. The smaller cohort sizes in earlier studies of ITH did not allow for examination of potential association with clinical outcomes. At present, this study design may be the only opportunity to examine the prognostic role of ITH at scale.

In conclusion, our study demonstrates that ITH is common in cancer, and that high levels of ITH impact survival outcomes negatively in many types of cancer. There are several possible explanations for this. Higher levels of heterogeneity increase the likelihood of a tumor harboring a subclonal population with a driver mutation that will be resistant to treatment. More generally, ITH may be a marker for a tumor's potential to evolve under selective pressure, and therefore its ability to grow in a fluctuating microenvironment. Additionally, high ITH may, in some cases, be the result of a tumor escape from the immunoediting processes that otherwise eliminate tumor cells or constrain clonal evolution.

In this study, the negative prognostic impact of ITH was a general feature of these tumors' biology, rather than attributable to a tumor's response to specific treatments. Indeed, ITH seems to be more than a measurable biologic process; our data indicate that it is a major obstacle to successful treatment of a patient's cancer. Ultimately, effective precision oncology will require the ability to target not only altered driver genes, but also the broad processes of tumor evolution. An improved understanding of the characteristics and consequences of ITH will be a necessary first step to targeting tumor evolution, whether with targeted therapy, immunotherapy, or a combination of multiple modalities.

## MATERIALS AND METHODS

### Data Sources

We analyzed data from genomics studies of solid tumors performed by The Cancer Genome Atlas Network (TCGA), using Affymetrix SNP6 array data for tumor and normal samples, clinical data, and exome sequencing data in level 3 curated MAF files. We report the results of all datasets analyzed. This study adheres to the REMARK (Reporting Recommendations for Tumor Marker Prognostic Studies) reporting guidelines [23]. A completed REMARK checklist is provided as Supplementary Table 10.

### Computational tools and workflow

SNP6 arrays were processed together, quantile-normalized, and median-polished using Affymetrix Power Tools. Allele-specific intensities were determined with the bird-seed algorithm, and then segmentation performed with allele-specific piecewise constant fitting (ASPCF). We used ASCAT 2.1 [24] to generate allele-specific copy number segmented information and tumor purity data. To infer subclonal populations, we used PyClone 0.12.7 [14]. For each non-synonymous single nucleotide variant called by TCGA, we input reference allele and variant allele read counts into PyClone. At each region, we additionally specified the copy number of the major and minor allele and estimates of tumor purity, both derived from ASCAT. PyClone output data are clustered using a Dirichlet process, to generate the number of mutation clusters in each sample. For the purposes of this study with a clinical endpoint, we considered the most biologically relevant clonal expansions as those defined by multiple mutations and therefore only included as SCPs clusters containing ≥2 unique mutations.

### Multivariable models

We used Cox multivariable regression after testing the proportional hazards assumption. The prognostic value of ITH was first examined in the discovery dataset, HNSC, given prior findings suggestive of such an association [22, 28]. The current analysis revealed that SCP > 4, corresponding to z-score (of log-transformed SCP) +1.75, maximized prognostic value in HNSC. We then applied the same threshold for high ITH (SCP > 4 ≈ log SCP z-score +1.75) in the additional cancer types. In almost all cancers, SCP > 4 also approximated z-score +1.75, and this cutpoint was used. For BRCA and KIRC, SCP > 4 did not approximate z-score +1.75, so z-score alone was used, corresponding to SCP>2 in BRCA and SCP>5 in KIRC. This was due to the distributions of SCPs in BRCA and KIRC being skewed differently than other cancers (Supplementary Table 2) (e.g. <1% of BRCA tumors had >4 SCPs). To rule out the possibility that the prognostic significance of ITH in BRCA and KIRC might be due to the choice of different SCP numerical thresholds, we ran additional analyses with multiple alternative cutpoints for high ITH. We found that high ITH remained significantly prognostic at each of the alternate SCP thresholds, indicating that the impact of ITH is robust and not limited to the specific SCP cutpoints (Supplementary Figure 9A-B).

### Immune populations

We used an *in silico* approach for the decomposition of immune cell populations in bulk mRNA-sequenced tumors using ssGSEA, methodological details of which have been reported separately [32]. The associations between immune infiltration, T cell infiltration, and interferon gamma signaling, and tumor heterogeneity were modeled with bivariate logistic regression controlling for cancer type.

## SUPPLEMENTARY MATERIAL FIGURES AND TABLES


