# Supplementary material for: Pan-cancer analysis of intratumor heterogeneity as a prognostic determinant of survival
Source: Oncotarget. 2016 Jan 28;7(9):10051–63. doi: 10.18632/oncotarget.7067 (PMC4891103; doi:10.18632/oncotarget.7067)
Supplement: Supplementary file 1 [file oncotarget-07-10051-s001.pdf]

Pan-cancer analysis of intratumor heterogeneity as a prognostic determinant of survival

Supplementary Material

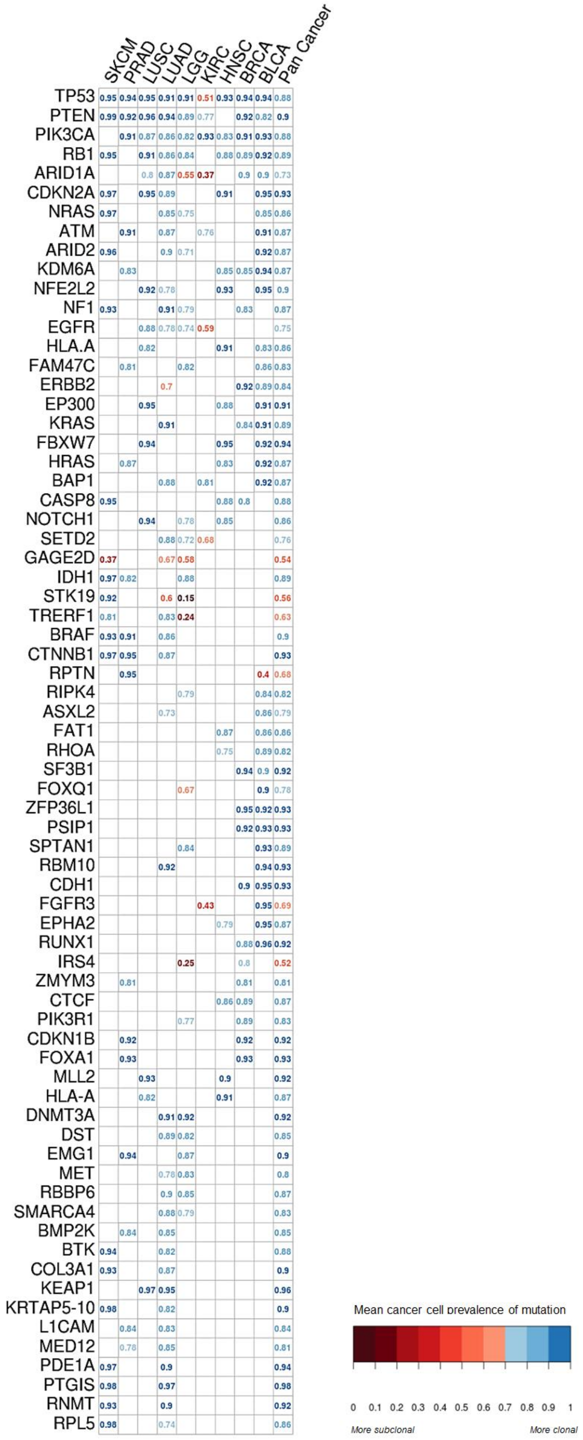

Supplementary Figure 1. Spectrum of (sub)clonality among recurrently mutated (MutSig q<0.10) genes in 9 cancer types. Each cell depicts the mean cancer cell prevalence of the gene mutation in that cancer type, and is colored by the mean cellular prevalence of the mutation in that cancer type.

HNSC

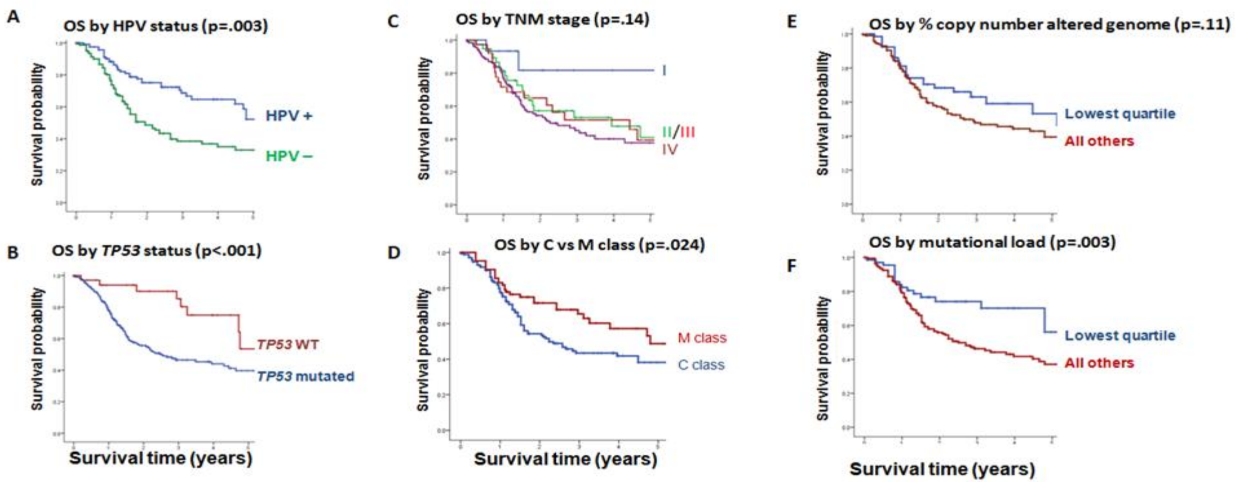

Supplementary Figure 2. Prognostic factors in the discovery dataset, head and neck cancer (HNSC). Kaplan-Meier curves showing overall survival based on (A) HPV status, (B) *TP53* gene mutation status, (C) pathologic tumor-node-metastasis (TNM) stage, (D) predominance of copy number alteration (C class) or mutational alteration (M class) as described by Ciriello et al [1], (E) percentage of genome harboring copy number alteration (log_2 copy number >0.2 or <-0.2), (F) mutational load (number of somatic non-synonymous mutations). P-values reflect comparisons made with the log-rank test.

HNSC

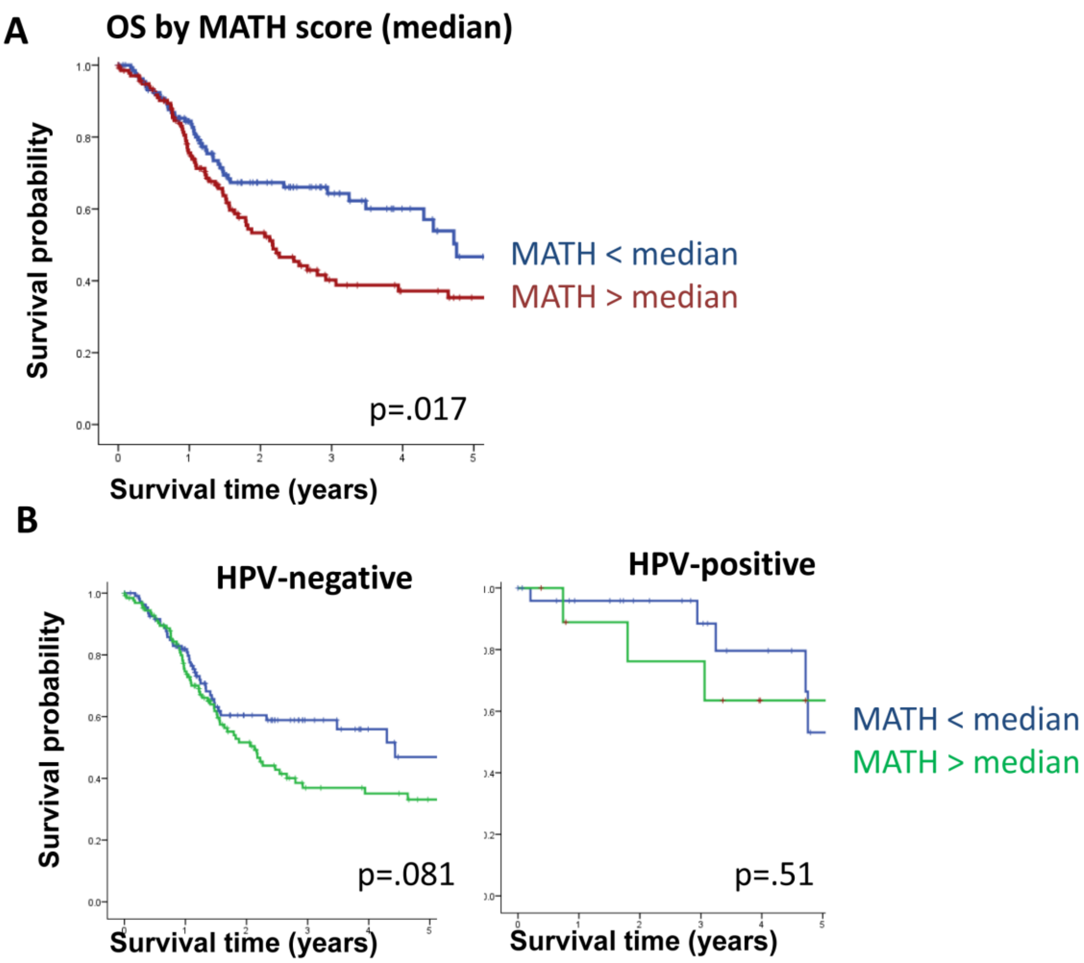

Supplementary Figure 3. Additional factors in the discovery dataset, head and neck cancer (HNSC). (A) MATH score as described by Mroz et al [2], and (B) MATH score stratified by HPV status. Comparisons were made with the log-rank test.

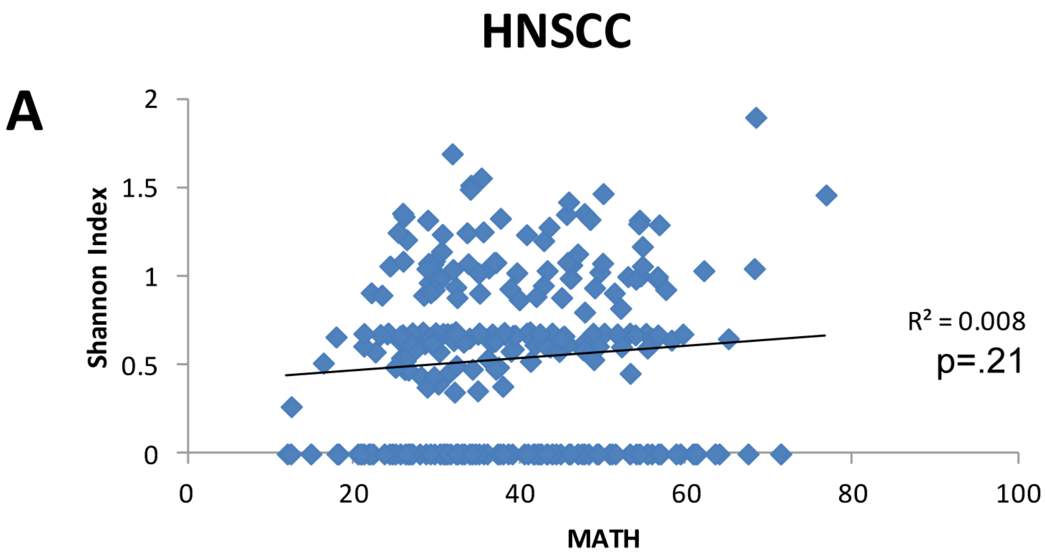

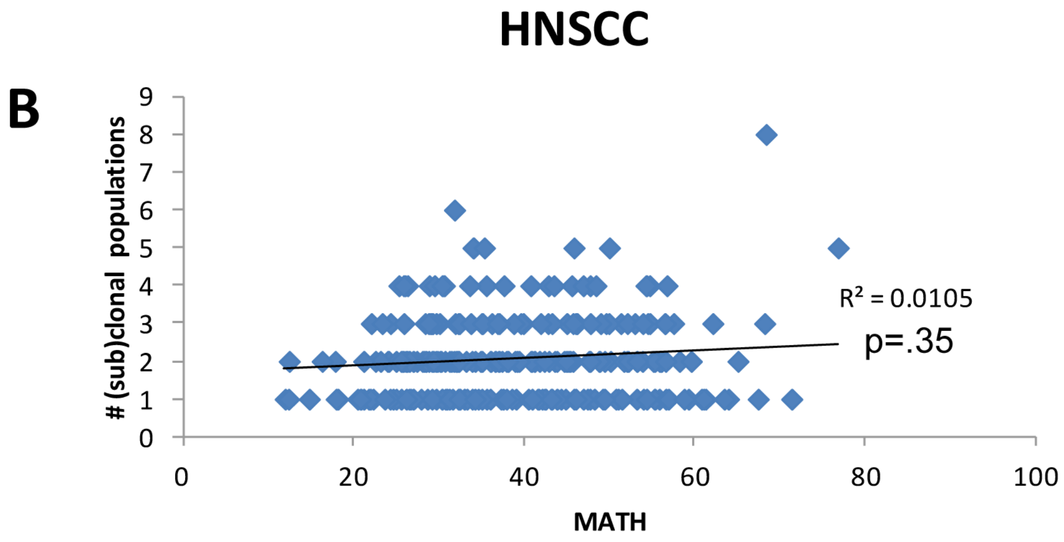

Supplementary Figure 4. Comparison of MATH score and other measures of intratumor heterogeneity, in head and neck cancer (HNSC).

(A) Scatterplot showing correlation between MATH and Shannon Index, a measure of intratumor heterogeneity. (B) Scatterplot showing correlation between MATH and the number of (sub)clonal populations in each tumor. Correlations are expressed as r^2 values and p value tested with Spearman non-parametric correlation.

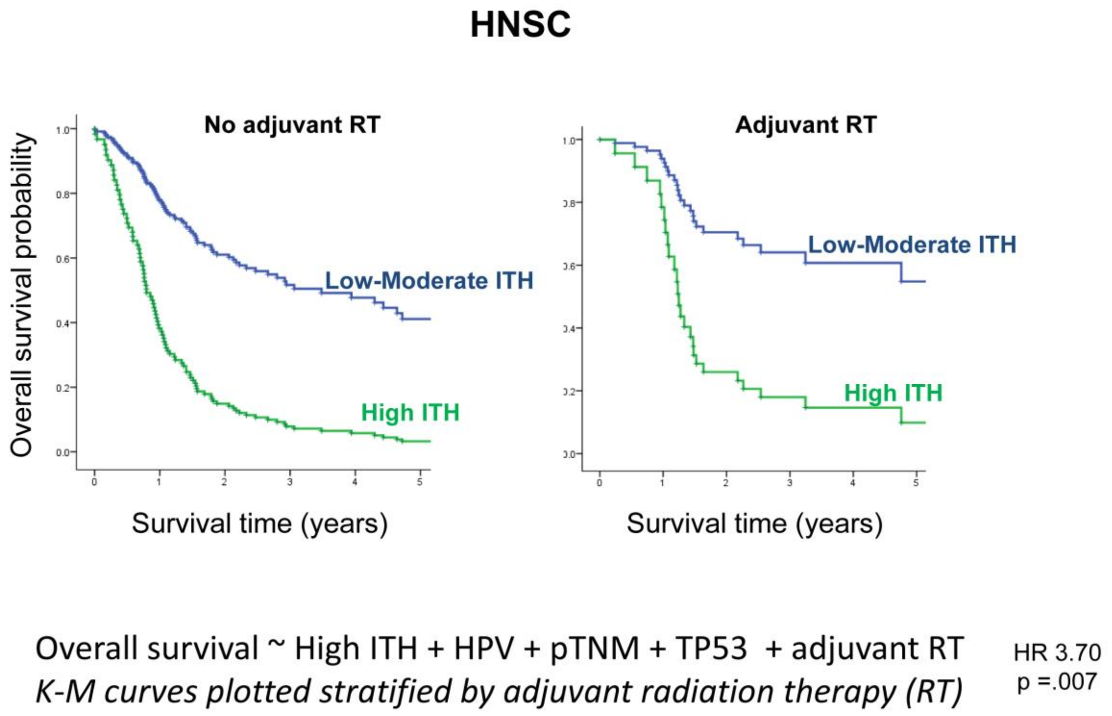

Supplementary Figure 5. Effect of intratumor heterogeneity on survival in head and neck cancers (HNSC), adjusting for treatment with adjuvant radiation therapy. Survival curves are plotted for low ITH and high ITH cases, at the mean of all other covariates (HPV status, TNM stage, TP53 status, and adjuvant radiation), and stratified for radiation vs. no radiation. In the Cox regression model, high ITH retained a significant association with poorer overall survival (HR = 3.70, p = .007). RT, radiation therapy.

LGG

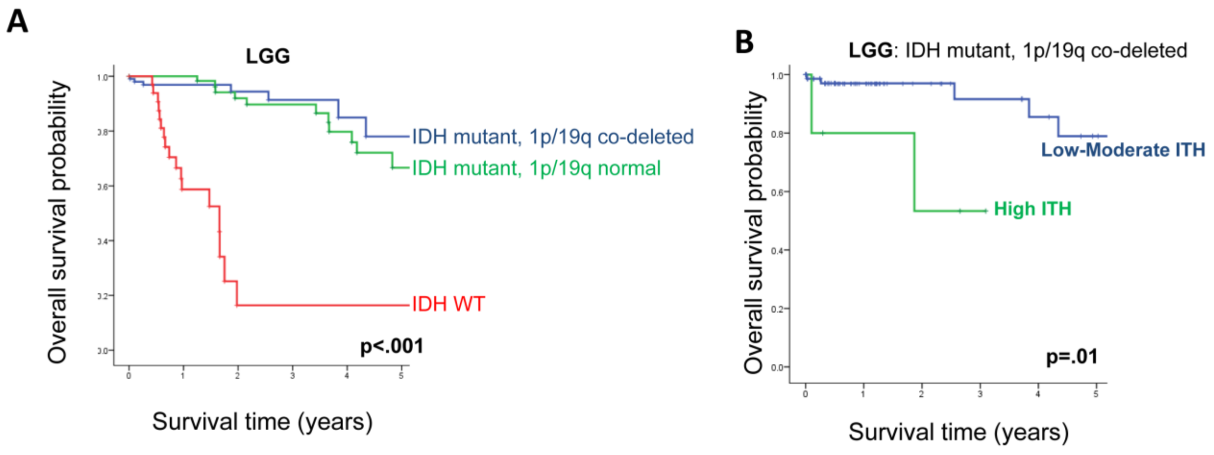

Supplementary Figure 6. Survival by molecular subtype and intratumor heterogeneity (ITH) in lower grade glioma (LGG). Kaplan-Meier curves for survival by molecular subtype, and for low ITH vs. high ITH in the IDH mutant, 1p/19q co-deleted subgroup. Comparisons made with the log-rank test.

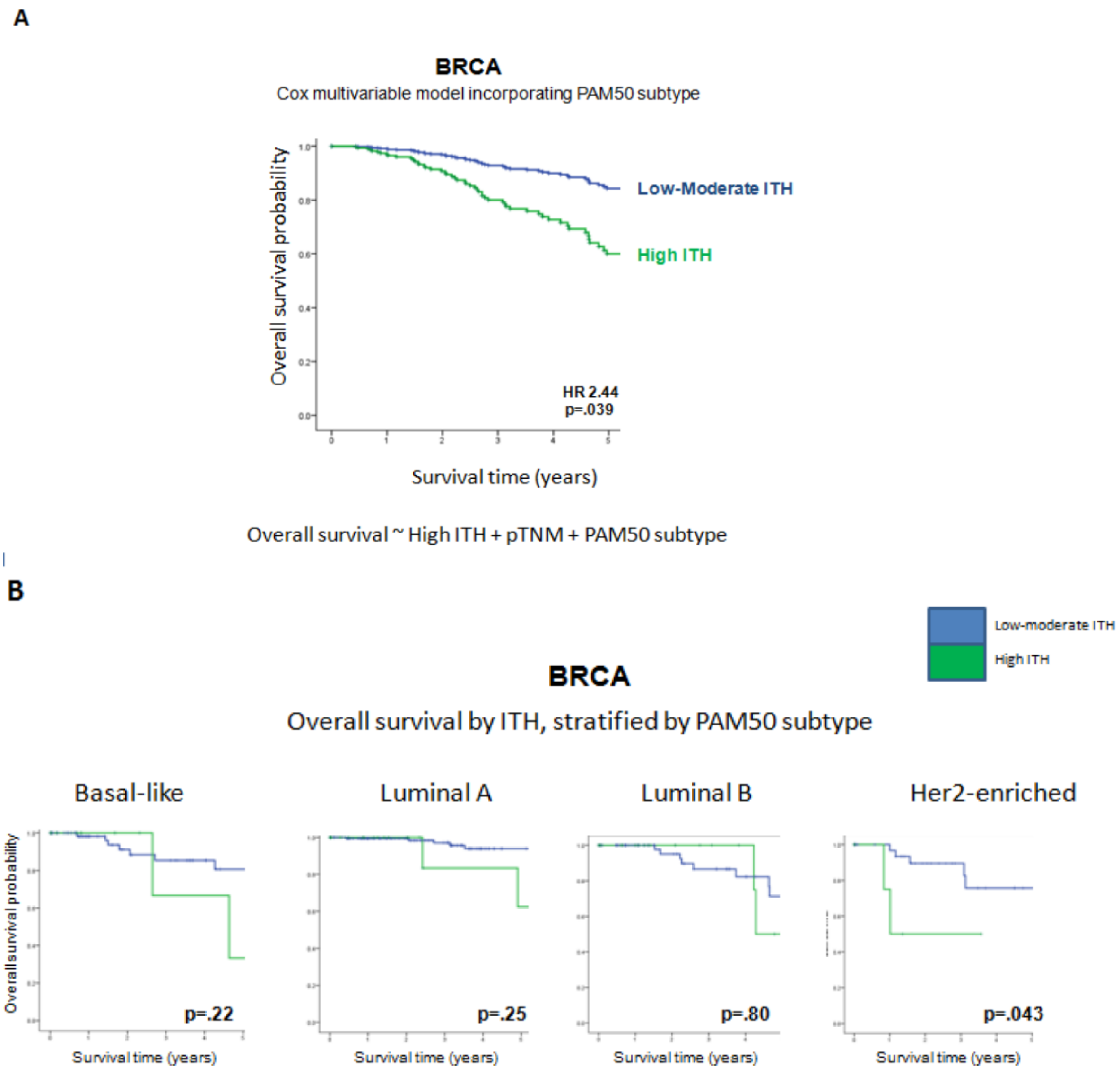

Supplementary Figure 7. Survival by molecular subtype and intratumor heterogeneity (ITH) in breast carcinoma (BRCA). (A) Cox regression model for overall survival by ITH, adjusting for pathologic TNM stage, and PAM50 molecular subtype. The survival curve is plotted by ITH status, at the mean of all other covariates. (B) Kaplan-Meier curves of overall survival by ITH status, for each molecular subtype of breast cancer.

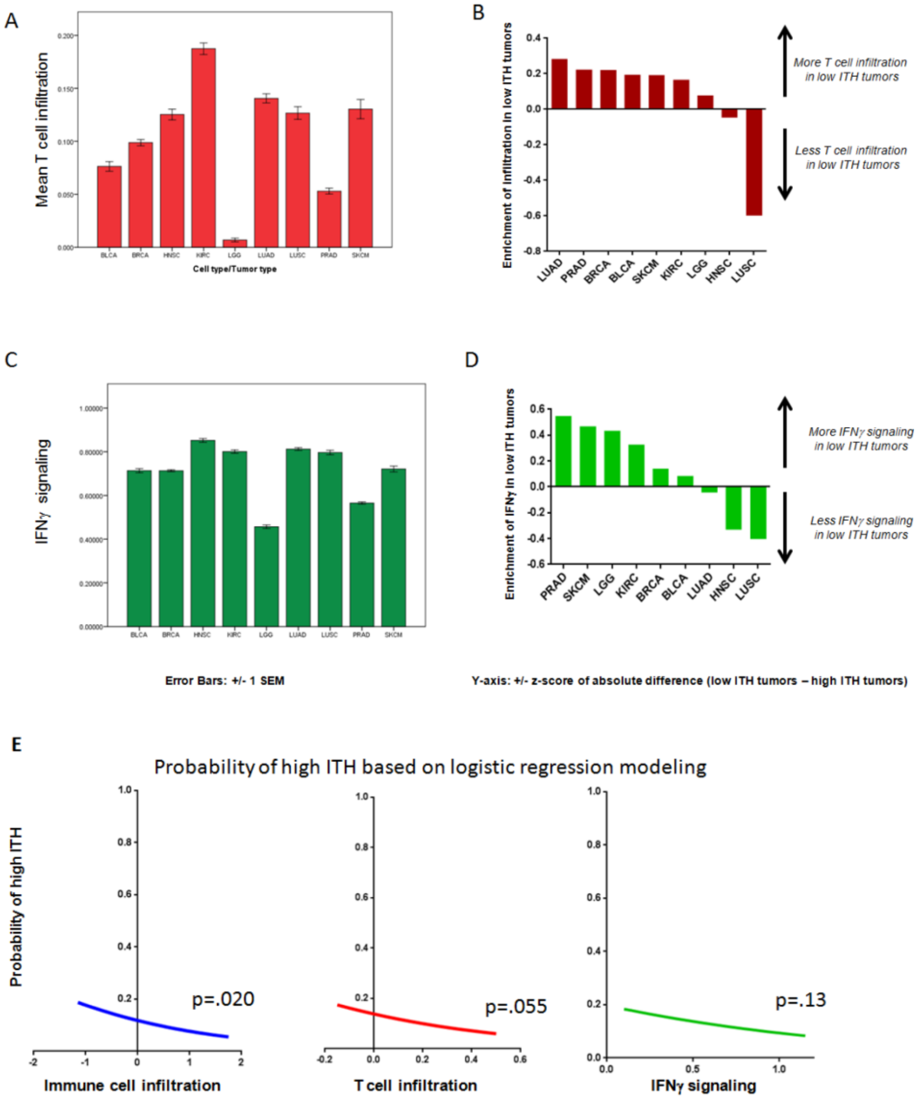

Supplementary Figure 8. Levels of T cell infiltration, and enrichment of IFNγ signaling, in 9 cancer types, and association with levels of intratumor heterogeneity (ITH). (A) Mean levels of RNAseq-derived levels of T cell infiltration by cancer type. (B) Column graph showing enrichment for T cell infiltration in tumors with low ITH, by cancer type. The y-axis represents the z-score of absolute increase, or decrease, in T cell infiltration in low ITH tumors. (C) Mean levels of IFNγ signaling enrichment, as determined by single sample Gene Set Enrichment Analysis in RNAseq data. (D) Column graph showing enrichment for IFNγ signaling in tumors with low ITH, by cancer type. The y-axis represents the z-score of absolute increase, or decrease, in enrichment of the IFNγ gene set in low ITH tumors. (E) Results of the logistic regression model of immune markers and ITH, showing the probability a tumor will have high ITH, based on immune markers: overall immune infiltrate, T cell infiltrate, and IFNγ signaling. P-values represent the significance of the covariate shown in the regression model, adjusting for cancer type.

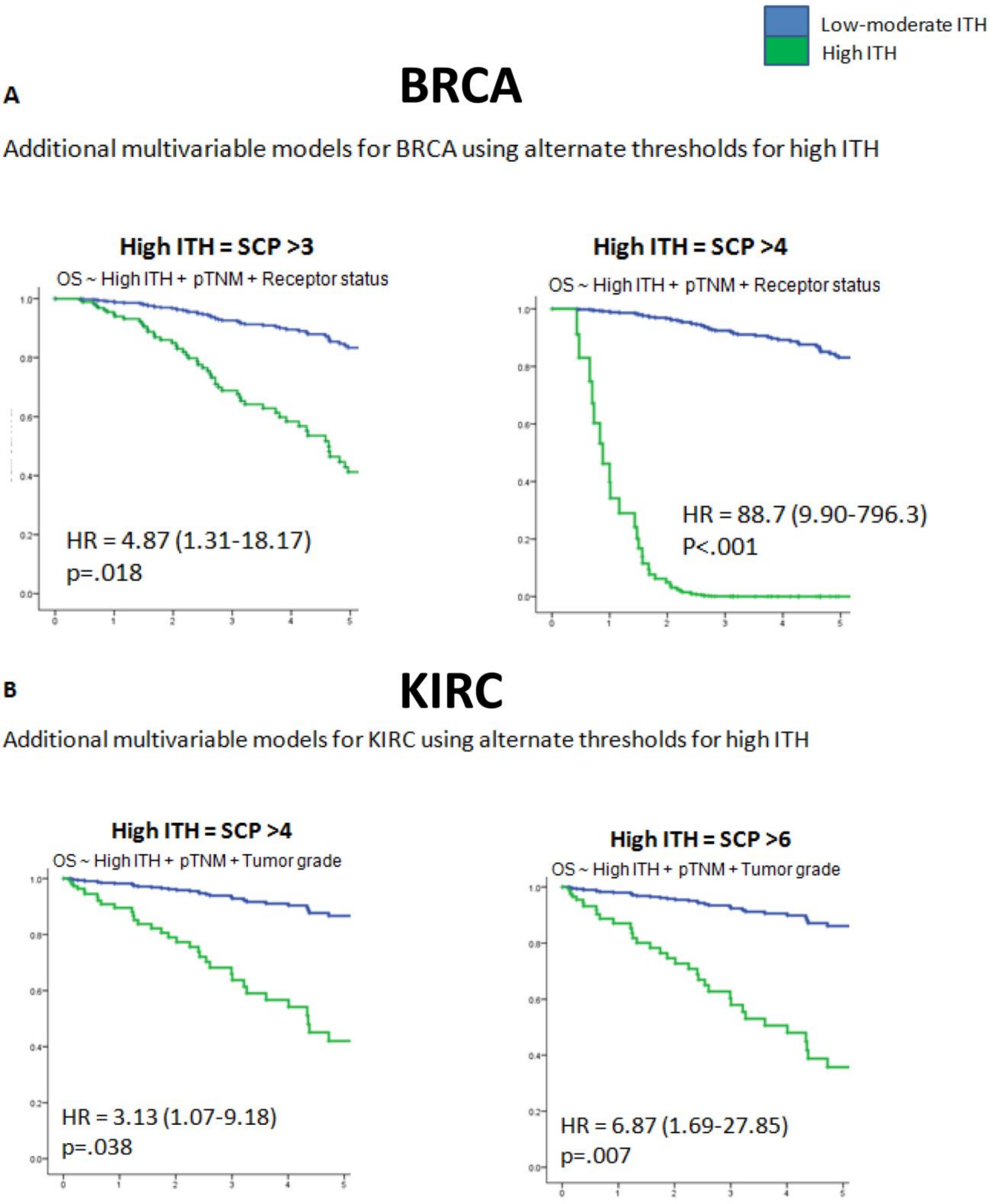

Supplementary Figure 9. Additional multivariable models for BRCA and KIRC, using alternate SCP thresholds to define ITH. Across cancers, high ITH was defined as tumors with (sub)clonal populations (SCP) SCP > 4 or SCP z-score +1.75. Because BRCA and KIRC were outliers compared to other cancers, in

terms of skewness of ITH (with BRCA having the lowest degree of ITH, and KIRC the highest), SCP > 4 did not approximate SCP z-score +1.75 in these cancers. Therefore, the z-score threshold alone was used, corresponding to cutpoints of SCP>2 for BRCA and SCP>5 for KIRC. In order to ensure that this choice of cutpoints was not responsible for the observed prognostic value of high ITH in BRCA and KIRC, we performed additional multivariable Cox regression analyses, instead using (A) SCP>3 and SCP>4 for BRCA, and (B) SCP>4 and SCP > 6 for KIRC, to show that the prognostic value of high ITH was robust across different cutpoints. These survival curves represent probabilities from the Cox regression model, plotted at the mean of all other covariates, as in Figure 3C. P-values represent the significance of the covariate high ITH, adjusting for all other terms in the model.

Supplementary Table 1
Total (sub)clonal populations, pan-cancer

SCPs	Number	Percentage
1	1394	41.4%
2	1170	34.8%
3	514	15.3%
4	174	5.2%
5	74	2.2%
6	26	0.8%
7	8	0.2%
8	3	0.1%
9	1	0.0%
Total	**3364**	**100.00%**

Supplementary Table 2
Shannon Index of analyzed tumors per cancer type

Cancer Type	Mean	Median
BLCA	2.04	2
BRCA	1.47	1
HNSC	2.08	2
KIRC	2.84	2
LGG	1.95	2
LUAD	2.36	2
LUSC	2.09	2
PRAD	1.86	2
SKCM	2.16	2
**Total**	**1.96**	**2**

Supplementary Table 3
Head and neck squamous cell carcinoma (HNSC):
correlations between MATH and other genetic measures

	Correlation (Spearman)	p value
**MATH vs ITH (number of SCPs)**	0.056	0.35
**MATH vs ITH (Shannon Index)**	0.075	0.21
**MATH vs copy number altered genome**	0.345	<.001
**MATH vs mutational load**	0.113	0.061

Supplementary Table 4
Association between ITH (number of SCPs) and overall survival in HNSC

Threshold for SCP (adjusted for HPV)	HR	95%CI	p
**SCP > 1**	1.23	1.02-1.49	0.033
**SCP > 2**	1.1	0.88-1.64	0.627
**SCP > 3**	1.28	0.74-2.20	0.376
**SCP > 4**	2.91	1.16-7.29	0.022

Supplementary Table 5
Multivariable modeling for OS in HNSC, incorporating ITH and other covariates

Supplementary Table 5A. Multivariable model in HNSC

Initial model	Final model	HR	95% LCI	95% UCI	p
High ITH	High ITH	3.75	1.43	9.84	0.007
HPV-negative	HPV-negative	2.51	1.13	5.59	0.024
Stage (pTNM)	Stage (pTNM)	-	-	-	0.304
TP53 mutation	TP53 mutation	1.55	1.03	2.33	0.038
C vs M class					
Copy number altered genome					
Mutational load					
**Log Likelihood = 1132.6 (p=.003)**	**Log Likelihood = 1171.4 (p=.001)**				

Supplementary Table 5B. Substitute MATH for High ITH

Covariates	HR	95% LCI	95% UCI	p
MATH (median)	0.87	0.72	1.05	0.15
HPV-negative	2.04	0.94	4.43	0.07
Stage (pTNM)	-	-	-	0.43
TP53 mutation	1.47	0.97	2.23	0.068

Supplementary Table 5C. Substitute Mutational Load for High ITH

Covariates	HR	95% LCI	95% UCI	p
Mutational Load	0.79	0.61	1.03	0.08
HPV-negative	1.88	0.86	4.09	0.12
Stage (pTNM)	-	-	-	0.56
TP53 mutation	1.52	1.01	2.28	0.046
**Add Mutational Load into Cox Model for OS (in addition to ITH)**				
High ITH	4.08	1.55	10.75	0.005
Mutational Load	1.00	0.998	1.001	0.276
HPV-negative	2.62	1.17	5.86	0.019
Stage (pTNM)	-	-	-	0.33
TP53 mutation	1.53	1.02	2.31	0.04

Supplementary Table 5D. Add Adjuvant RT into Cox model for OS (in addition to ITH)

Covariates	HR	95% LCI	95% UCI	p
High ITH	3.71	1.42	9.68	0.007
HPV-negative	2.41	1.08	5.35	0.031

Stage (pTNM)	-	-	-	0.13
TP53	1.57	1.05	2.37	0.03
Adjuvant RT treatment	0.61	0.39	0.96	0.032

Supplementary Table 6
Correlation of ITH with mutational load

Supplementary Table 6A. Correlation of ITH (number of SCPs) with mutational load (non-synonymous SNVs)

Cancer type	Spearman correlation	p value
BLCA	**0.195**	**<.001**
BRCA	**0.283**	**<.001**
HNSC	**0.251**	**<.001**
KIRC	**0.238**	**0.001**
LGG	**0.288**	**<.001**
LUAD	0.017	0.732
LUSC	0.077	0.331
PRAD	**0.178**	**<.001**
SKCM	**0.349**	**<.001**

Supplementary Table 6B. Addition of mutational load into Cox regression models for survival in cancer types where mutational load and ITH were correlated
(additional models were run in cancer types where ITH was prognostic)

	p value for ITH	p value for mutational load
BRCA	**0.013**	0.525
HNSC	**0.005**	0.276
KIRC	**0.011**	0.175
LGG	**0.01**	0.879
PRAD	**0.018**	0.976
SKCM	**0.038**	0.296

Supplementary Table 7

Correlation of ITH with patient age.

Supplementary Table 7A. Correlation of ITH (number of SCPs) with patient age at diagnosis

Cancer type	Correlation	p value
BLCA	-0.094	0.267
BRCA	0.046	0.17
HNSC	0.049	0.42
KIRC	0.069	0.35
**LGG**	**0.15**	**0.001**
LUAD	-0.039	0.43
LUSC	-0.059	0.46
PRAD	0.06	0.24
SKCM	0.12	0.082

Supplementary Table 7B.
Add age into Cox regression model for overall survival in LGG

Covariate	HR	95% LCI	95% UCI	p
High ITH	10.19	2.02	51.5	0.005
IDH, 1p-19q status	-	-	-	<.001
Age	1.068	1.037	1.099	<.001

Supplementary Table 8.

Binary logistic regression model for immune factors associated with high ITH (pan-cancer)

High ITH ~ Immune Infiltration + Cancer Type

Covariate	OR	p value
Immune Infiltration	0.62	0.020
Cancer Type	-	<.001

Covariate	OR	p value
T cell Infiltration	0.16	0.055
Cancer Type	-	<.001

Supplementary Table 9

Addition of immune infiltration data into multivariable survival models

Supplementary Table 9A. KIRC: Add immune infiltration scores into Cox regression model for OS

Covariate	HR	95% LCI	95% UCI	p
High ITH	6.16	2.7	14.03	<.001
Stage (pTNM)	-	-	-	0.088
Grade	-	-	-	0.039
Immune infiltration	0.74	0.25	2.15	0.58

Covariate	HR	95% LCI	95% UCI	p
High ITH	6.47	2.87	14.58	<.001
Stage (pTNM)	-	-	-	0.09
Grade	-	-	-	0.069
T cell infiltration	0.79	0.34	1.83	0.59

Supplementary Table 9B. HNSC: Add immune infiltration scores into Cox regression model for OS

Covariate	HR	95% LCI	95% UCI	p
High ITH	1.39	1.09	1.76	0.008
HPV status	2.12	0.93	4.8	0.073
Stage (pTNM)	-	-	-	0.32
TP53 status	1.46	0.97	2.21	0.07
Immune infiltration	0.73	0.47	1.12	0.15

Covariate	HR	95% LCI	95% UCI	p
High ITH	1.41	1.11	1.79	0.005
HPV status	2.07	0.92	4.64	0.077
Stage (pTNM)	-	-	-	0.38
TP53 status	1.39	0.92	2.11	0.12
T cell infiltration	0.029	0.002	0.389	0.007

Supplementary Table 10. REMARK (Reporting Recommendations for Tumor Marker Prognostic Studies) checklist.

	Item to be reported	Page no.
**INTRODUCTION**		
1	State the marker examined, the study objectives, and any pre-specified hypotheses.	3
**MATERIALS AND METHODS**		
Patients		
2	Describe the characteristics (e.g., disease stage or co-morbidities) of the study patients, including their source and inclusion and exclusion criteria.	3,11
3	Describe treatments received and how chosen (e.g., randomized or rule-based).	3,11
Specimen characteristics		
4	Describe type of biological material used (including control samples) and methods of preservation and storage.	3,11
Assay methods		
5	Specify the assay method used and provide (or reference) a detailed protocol, including specific reagents or kits used, quality control procedures, reproducibility assessments, quantitation methods, and scoring and reporting protocols. Specify whether and how assays were performed blinded to the study endpoint.	11,12,13,14,15
Study design		
6	State the method of case selection, including whether prospective or retrospective and whether stratification or matching (e.g., by stage of disease or age) was used. Specify the time period from which cases were taken, the end of the follow-up period, and the median follow-up time.	3,11
7	Precisely define all clinical endpoints examined.	5,13,14
8	List all candidate variables initially examined or considered for inclusion in models.	5,6,13,14
9	Give rationale for sample size; if the study was designed to detect a specified effect size, give the target power and effect size.	3,11
Statistical analysis methods		
10	Specify all statistical methods, including details of any variable selection procedures and other model-building issues, how model assumptions were verified, and how missing data were handled.	13,14
11	Clarify how marker values were handled in the analyses; if relevant, describe methods used for cutpoint determination.	13,14
**RESULTS**		
Data		
12	Describe the flow of patients through the study, including the number of patients included in each stage of the analysis (a diagram may be helpful) and reasons for dropout. Specifically, both overall and for each subgroup extensively examined report the numbers of patients and the number of events.	3,11
13	Report distributions of basic demographic characteristics (at least age and sex), standard (disease-specific) prognostic variables, and tumor marker, including numbers of missing values.	3,11
Analysis and presentation		
14	Show the relation of the marker to standard prognostic variables.	6,7
15	Present univariable analyses showing the relation between the marker and outcome, with the estimated effect (e.g., hazard ratio and survival probability). Preferably provide similar analyses for all other variables being analyzed. For the effect of a tumor marker on a time-to-event outcome, a Kaplan-Meier plot is recommended.	6
16	For key multivariable analyses, report estimated effects (e.g., hazard ratio) with confidence intervals for the marker and, at least for the final model, all other variables in the model.	Table 1, page 6

17	Among reported results, provide estimated effects with confidence intervals from an analysis in which the marker and standard prognostic variables are included, regardless of their statistical significance.	Table1
18	If done, report results of further investigations, such as checking assumptions, sensitivity analyses, and internal validation.	6,7,8
**DISCUSSION**		
19	Interpret the results in the context of the pre-specified hypotheses and other relevant studies; include a discussion of limitations of the study.	8,10
20	Discuss implications for future research and clinical value.	9,10

MATERIALS AND METHODS

Data Sources

We analyzed data from genomics studies of solid tumors performed by The Cancer Genome Atlas Network (TCGA), for which 3 types of complete data were available for ≥100 tumors: 1) SNP 6.0 array copy number, 2) single nucleotide variant calls and read counts (as reported by the TCGA in .MAF files), and 3) clinical patient and tumor data. We did not include cancer sites if cohort sizes were <100, if requisite details for the 3 types of data were not publicly available (e.g. read count data were not completely available for colorectal, ovarian, uterine, or gastric tumors), if the TCGA cancer study had not been published, or if the cancer type exhibited insufficient spread in survival times to make modeling of prognostic features likely to be feasible (e.g., glioblastoma or papillary thyroid carcinoma). Affymetrix SNP6 array data for tumor and normal samples for each cancer type studied were downloaded from the TCGA Data portal on 1/28/15. Clinical data were downloaded on 6/3/2015. Level 3 curated MAF files were downloaded from the Broad Institute Firehose pipeline on 6/3/2015. Mutations were filtered to variants with at least 7 reads for subsequent analysis. Only single nucleotide variants were considered.

We analyzed data for nine cancer sites: bladder urothelial carcinoma (BLCA, n=359), breast invasive carcinoma (BRCA, n=878), head and neck squamous cell carcinoma (HNSC, n=280), clear cell carcinoma of the kidney (KIRC, n=189), lower grade glioma (LGG, n=484), lung adenoncarcinoma (LUAD, n=425), lung squamous cell carcinoma (LUSC, n=178), prostate adenocarcinoma (PRAD, n=389), and melanoma (SKCM, n=201). We report the results of all datasets analyzed. This study adheres to the REMARK (Reporting Recommendations for Tumor Marker Prognostic Studies) reporting guidelines [3]. A completed REMARK checklist is provided as **Supplementary Table 10**.

Clinical data definitions

Molecular subtypes were defined based on TCGA data for HNSC (HPV status), BRCA (ER, PR and Her2 receptor status, and PAM50 expression subtype), and LGG (*IDH* and 1p/19q status). For all cancer types other than prostate carcinoma, the survival outcome modeled was overall survival (OS), where events are defined as death of any cause based on the Vital Status data field, and time in days. For prostate cancer, where deaths were rare in the cohort during available followup, we used relapse-free survival (RFS), where events were recurrence or death of any cause. All Kaplan-Meier survival curves are plotted with the x-axis ranging from 0-5 years.

Computational tools and workflow

SNP6 array data was used to determine copy number information. For each individual cancer type, arrays were processed together, quantile-normalized, and median-polished using Affymetrix Power Tools. Allele-specific intensities were determined with the bird-seed algorithm, and then segmentation performed with allele-specific piecewise constant fitting (ASPCF) for the Log R ratio and B allele frequency tracks. We used ASCAT 2.1 [4] to generate allele-specific copy number segmented information. ASCAT performs a grid-search over possible purity and ploidy combinations to identify values that best fit SNP6 intensity data, and then subsequently estimates the allele-specific copy number

for each segment. Samples that were unable to be fit by ASCAT were not used for further analysis. We validated the ASCAT copy number-based estimates of tumor purity by using an orthogonal technique, ESTIMATE, which uses expression data to infer tumor purity[5]. The two techniques for estimating tumor purity were highly concordant (for HNSC, Spearman rho=-.406, $p<1x10^{-7}$).

To infer subclonal populations, we used PyClone 0.12.7 [6]. PyClone estimates the cellular prevalence of a mutation by modeling tumors as comprised of admixed 1) normal non-tumor cells, 2) cancer cells lacking a specified mutation, and 3) cancer cells harboring a specified mutation. The cells within each population are modeled as having identical genotype. If one assumes that no site is mutated more than once, and that mutations do not revert or disappear, this model implies that mutations occurring at the same point in time during clonal evolution are present at similar cellular prevalences. The cellular prevalence rates are then clustered into subclonal populations (SCPs). A theoretical limitation of PyClone is that the copy number prior impacts the final result; therefore, high degrees of copy number heterogeneity would not be accounted for.

For each non-synonymous single nucleotide variant called by TCGA, we input reference allele and variant allele read counts into PyClone. At each region, we additionally specified the copy number of the major and minor allele and estimates of tumor purity, both derived from ASCAT. PyClone was run using the infinite beta binomial mixture model fit with 10,000 iterations and default priors. The output data are then clustered with a burn-in of 1000, using a Dirichlet process, to generate the number of mutation clusters in each sample. Post-processing was performed in PyClone to plot similarity matrices and cellular prevalence density plots for manual inspection.

We confirmed that our SCP data were similar to the distribution of previously reported SCP data in an independent cohort of breast cancers also analyzed with PyClone [7], with minor differences in results accounted for differences in the minimum SNV number per SCP. For the purposes of this study with a clinical endpoint, we considered the most biologically relevant clonal expansions as those defined by multiple mutations and therefore only included as SCPs clusters containing ≥2 unique mutations. We classified tumors as having high or low-moderate ITH based on the number of SCPs (see below, *Multivariable Models*). Mutations with q value <0.10 as determined by MutSigCV as reported by TCGA in Firehose data were included in the landscape of clonal vs. subclonal events per cancer type.

PyClone-inferred SCP number has been demonstrated to have high concordance with data from single-cell sequencing [8]. As further confirmation of our analytic process, we also analyzed the first implemented dataset (HNSC) with an independent tool that uses a different mathematical methodology to infer SCPs. EXPANDS uses total tumor copy number and somatic mutation allele frequency to infer cancer cell prevalence, followed by clustering via information divergence to identify SCPs [9]. The numbers of SCPs generated by the two methods were examined with linear correlation. Despite having two different methodologies, data generated by our workflow and by EXPANDS were highly concordant ($p=3.01 x10^{-4}$).

It is anticipated that this approach may underestimate the extent of ITH, because copy number and mutational data are derived from single regions or bulk tumor samples. Regional variation and subclonal

populations below the resolution of these techniques would not reliably be detected without more extensive multi-region sequencing such as single-cell techniques. Similarly, SCPs present at low abundance are known to require very deep sequencing coverage to be reliably identified [10].

Quantitative measures

We used the PyClone output of number of clusters to represent the number of subclonal or clonal populations (SCPs) in each tumor. Tumors with a given number of SCPs can still differ in heterogeneity, depending on the abundance of each SCP [11]. Tumors with coexisting SCPs at similar proportional abundances will tend to have higher entropy (or diversity), in contrast to tumor SCPs with very unequal abundances. One method that has been used to quantify diversity in tumor cell populations is the Shannon Index (H'):

$$H' = -\sum_{i=1}^{R} p_i \ln p_i$$

R represents the number of SCPs, and p_i represents the mean cancer cell prevalence of the *i*th SCP. The Shannon Index was originally described as a measure of information content and has subsequently adopted in other studies for use representing species diversity or the degree of diversity in heterogeneous tumors [9, 11]. It can be considered a measure of the uncertainty in predicting which SCP a cancer cell belongs to. Computation in the context of SCPs requires the simplifying assumption that cancer cells belong to only one SCP.

Dispersion in mutation variant allele frequencies can be expressed using MATH (mutant allele tumor heterogeneity) [2]. This measure is calculated as the median absolute deviation of each somatic mutation's allelic fraction from the median allelic fraction for all mutations in the tumor, divided by the median variant allelic fraction. MATH would be expected to vary with local ploidy and loss of heterozygosity across genomic loci harboring mutations. For each cancer type, we examined the correlation of MATH to measures of ITH, both number of SCPs and Shannon Index, and other genomic features such as mutational load and copy number alteration, using ordinary least squares regression and Spearman non-parametric correlation.

Multivariable models

The prognostic value of ITH was first examined in the discovery dataset, HNSC, given prior findings suggestive of such an association [2]. The log-rank test was used to determine the association between ITH and overall survival, stratified by HPV status. We performed a sensitivity analysis to test the robustness of the association between high ITH and survival across a range of ITH thresholds. In all cases, the distinction is between "high ITH" and "low-moderate ITH" (ie, all others), with no intermediate cases excluded. This analysis revealed that SCP > 4, corresponding to z-score (of log-transformed SCP) +1.75, maximized prognostic value in HNSC. Clinical, pathologic and molecular covariates were entered into a proportional hazards regression model together with high ITH. Stepwise conditional elimination was used to build a parsimonious model based on likelihood ratios. The proportional hazards assumption was tested using the log negative log survival function.

After initial identification of an association between high ITH and survival outcome in HNSC, we then applied the identical threshold for high ITH (SCP > 4 ≈ log SCP z-score +1.75) in the additional cancer types. In almost all cancer types, SCP > 4 approximated z-score +1.75, as in HNSC, and this cutpoint was used. For BRCA and KIRC, SCP > 4 did not approximate SCP z-score +1.75, so z-score alone was used. This was due to the distributions of SCPs in these 2 cancer types being skewed differently than in other cancers. BRCA had the lowest mean level of ITH; KIRC, the highest (**Supplementary Table 2**). As a result, only 0.2% of BRCA cases would have SCP>4. SCP z-score +1.75 corresponded to SCP>2 in BRCA and SCP>5 in KIRC, and these cutpoints were therefore used to define high ITH with consistency. These thresholds for high ITH were all chosen prior to analysis, but we subsequently ran additional analyses for BRCA and KIRC, using multiple alternative cutpoints for high ITH (SCP>3 and SCP>4 for BRCA; SCP>4 and SCP>6 for KIRC), to determine whether the prognostic significance of high ITH was robust, and to rule out the possibility that the observed prognostic value of high ITH in BRCA and KIRC might have been due to the application of different SCP thresholds. We found that high ITH remained significantly prognostic at each of the alternate SCP thresholds, indicating that the impact of ITH is robust and not the result of the specific SCP numerical cutpoint selected. These data are shown in **Supplementary Figure 9A-B**.

Cox regression was used to determine the independent prognostic value for ITH in each cancer type, adjusting for other significant or clinically relevant covariates. Survival curves were separated for low-moderate ITH and high ITH (no intermediate cases were excluded), plotting cumulative hazard at the mean of all covariates in the model.

Measures of high ITH were examined for associations with multiple other clinical, pathologic and molecular covariates, including patient age, smoking history, tumor size, tumor (T) stage, metastatic status, prior treatment, mutational load per tumor, mutation of driver genes, and molecular subtype. Correlation (Spearman for non-parametric; Pearson for parametric) and chi-squared or Fisher exact tests were used for these analyses.

Immune populations

We used an *in silico* approach for the decomposition of immune cell populations in bulk mRNA-sequenced tumors, details of which have been reported separately [12]. This method applies expression-based gene signatures of immune cell populations [13] to individual tumor samples using single sample gene set enrichment analysis (ssGSEA) [14]. ssGSEA computes an overexpression score for a gene signature by comparing the ranks of the genes in the signature with the ranks of all other genes in the transcriptome. We characterized the degree of immune infiltration in 9 TCGA cancer cohorts by running ssGSEA with 24 expression-based immune gene signatures, comprising 509 genes in total. The input data, pan-cancer normalized gene-level RNA-seq datasets, were downloaded from the UC Santa Cruz Cancer Genomics Browser (https://genome-cancer.ucsc.edu/).

The overall score for immune infiltration includes macrophages, dendritic cell subsets [total, plasmacytoid, immature, activated], B cells, cytotoxic cells [CD8+ T cells and NK cells], eosinophils, mast cells, neutrophils, NK cell subsets [total, CD56bright, CD56dim], and T cell subsets [CD8+, T helper, T central and effector memory, Th1, Th2, and Treg cells]. This algorithm has been orthogonally validated

in samples studied with immunofluorescence staining, with high rates of concordance [12]. Measurements of tumor purity and stromal content in tumors correlate inversely with the degree of immune cell infiltrate. Interferon gamma signaling was scored with ssGSEA using the REACTOME interferon gamma geneset (http://www.broadinstitute.org/gsea/msigdb/cards/REACTOME_INTERFERON_GAMMA_SIGNALING). The interferon gamma geneset had minimal redundancy with the immune population genes (3/63 overlap with 509 immune genes). Mean immune cell infiltration scores were displayed, with T cell infiltration scores adjusted uniformly by +0.15 for ease of viewing.

The associations between immune infiltration, T cell infiltration, and interferon gamma signaling, and tumor heterogeneity were modeled with bivariate logistic regression (with ITH the dependent variable, categorized as low/monoclonal, or high), controlling for cancer type. Immune infiltration, T cell infiltration and interferon gamma signaling scores were all normally distributed. The difference in immune infiltration scores by low or high ITH was shown for each cancer type, by plotting the z-score of the absolute difference between mean immune infiltration score in low ITH tumors and high ITH tumors. The overall association between immune cell infiltration scores and ITH was depicted by plotting the modeled probabilities of a sample having high ITH, at the mean of all cancer types, across the full range of immune infiltration or interferon signaling scores.

REFERENCES

1. Ciriello G, Miller ML, Aksoy BA, Senbabaoglu Y, Schultz N and Sander C. Emerging landscape of oncogenic signatures across human cancers. Nature genetics. 2013; 45(10):1127-1133.
2. Mroz EA, Tward AD, Pickering CR, Myers JN, Ferris RL and Rocco JW. High intratumor genetic heterogeneity is related to worse outcome in patients with head and neck squamous cell carcinoma. Cancer. 2013; 119(16):3034-3042.
3. McShane LM, Altman DG, Sauerbrei W, Taube SE, Gion M, Clark GM and Statistics Subcommittee of the NCIEWGoCD. Reporting recommendations for tumor marker prognostic studies (REMARK). Journal of the National Cancer Institute. 2005; 97(16):1180-1184.
4. Van Loo P, Nordgard SH, Lingjaerde OC, Russnes HG, Rye IH, Sun W, Weigman VJ, Marynen P, Zetterberg A, Naume B, Perou CM, Borresen-Dale AL and Kristensen VN. Allele-specific copy number analysis of tumors. Proceedings of the National Academy of Sciences of the United States of America. 2010; 107(39):16910-16915.
5. Yoshihara K, Shahmoradgoli M, Martinez E, Vegesna R, Kim H, Torres-Garcia W, Trevino V, Shen H, Laird PW, Levine DA, Carter SL, Getz G, Stemke-Hale K, Mills GB and Verhaak RG. Inferring tumour purity and stromal and immune cell admixture from expression data. Nature communications. 2013; 4:2612.
6. Roth A, Khattra J, Yap D, Wan A, Laks E, Biele J, Ha G, Aparicio S, Bouchard-Cote A and Shah SP. PyClone: statistical inference of clonal population structure in cancer. Nature methods. 2014; 11(4):396-398.
7. Shah SP, Roth A, Goya R, Oloumi A, Ha G, Zhao Y, Turashvili G, Ding J, Tse K, Haffari G, Bashashati A, Prentice LM, Khattra J, Burleigh A, Yap D, Bernard V, et al. The clonal and mutational evolution spectrum of primary triple-negative breast cancers. Nature. 2012; 486(7403):395-399.
8. Eirew P, Steif A, Khattra J, Ha G, Yap D, Farahani H, Gelmon K, Chia S, Mar C, Wan A, Laks E, Biele J, Shumansky K, Rosner J, McPherson A, Nielsen C, et al. Dynamics of genomic clones in breast cancer patient xenografts at single-cell resolution. Nature. 2015; 518(7539):422-426.
9. Andor N, Harness JV, Muller S, Mewes HW and Petritsch C. EXPANDS: expanding ploidy and allele frequency on nested subpopulations. Bioinformatics. 2014; 30(1):50-60.
10. Cibulskis K, Lawrence MS, Carter SL, Sivachenko A, Jaffe D, Sougnez C, Gabriel S, Meyerson M, Lander ES and Getz G. Sensitive detection of somatic point mutations in impure and heterogeneous cancer samples. Nature biotechnology. 2013; 31(3):213-219.
11. Merlo LM, Shah NA, Li X, Blount PL, Vaughan TL, Reid BJ and Maley CC. A comprehensive survey of clonal diversity measures in Barrett's esophagus as biomarkers of progression to esophageal adenocarcinoma. Cancer prevention research. 2010; 3(11):1388-1397.
12. Senbabaoglu Y, Winer A, Gejman R, Liu M, Luna A, Ostrovnaya I, Weinhold N, Lee W, Sander C and Hakimi A. The landscape of T cell infiltration in human cancer and its association with antigen presenting gene expression. bioRxiv doi:101101/025908 2015.
13. Bindea G, Mlecnik B, Tosolini M, Kirilovsky A, Waldner M, Obenauf AC, Angell H, Fredriksen T, Lafontaine L, Berger A, Bruneval P, Fridman WH, Becker C, Pages F, Speicher MR, Trajanoski Z, et al. Spatiotemporal dynamics of intratumoral immune cells reveal the immune landscape in human cancer. Immunity. 2013; 39(4):782-795.
14. Barbie DA, Tamayo P, Boehm JS, Kim SY, Moody SE, Dunn IF, Schinzel AC, Sandy P, Meylan E, Scholl C, Frohling S, Chan EM, Sos ML, Michel K, Mermel C, Silver SJ, et al. Systematic RNA interference reveals that oncogenic KRAS-driven cancers require TBK1. Nature. 2009; 462(7269):108-112.
